# Clinical prediction rule for identifying older patients with toxigenic *clostridioides difficile* at the time of hospital admission

**DOI:** 10.1186/s12877-023-03808-2

**Published:** 2023-03-06

**Authors:** Ki-Byung Lee, Mina Lee, Jin Woong Suh, Kyung-Sook Yang, Youseung Chung, Jeong Yeon Kim, Sun Bean Kim, Jang Wook Sohn, Young Kyung Yoon

**Affiliations:** 1 Division of Infectious Diseases, Department of Internal Medicine, Korea University Anam Hospital, Korea University College of Medicine, 73, Inchon-ro, Seongbuk-gu, 02841 Seoul, Republic of Korea; 2 Infection Control Unit, Korea University Anam Hospital, Seoul, Republic of Korea; 3 Department of Biostatistics, Korea University College of Medicine, Seoul, Republic of Korea

**Keywords:** *Clostridioides difficile*, Clinical prediction rule, Risk factors, Active surveillance, Infection control

## Abstract

**Background:**

This study aimed to develop and validate a clinical prediction rule to screen older patients at risk of being toxigenic *Clostridioides difficile* carriers at the time of hospital admission.

**Methods:**

This retrospective case-control study was performed at a university-affiliated hospital. Active surveillance using a real-time polymerase chain reaction (PCR) assay for the toxin genes of *C. difficile* was conducted among older patients (≥ 65 years) upon admission to the Division of Infectious Diseases of our institution. This rule was drawn from a derivative cohort between October 2019 and April 2021 using a multivariable logistic regression model. Clinical predictability was evaluated in the validation cohort between May 2021 and October 2021.

**Results:**

Of 628 PCR screenings for toxigenic *C. difficile* carriage, 101 (16.1%) yielded positive findings. To establish clinical prediction rules in the derivation cohort, the formula was derived using significant predictors for toxigenic *C. difficile* carriage at admission, such as septic shock, connective tissue diseases, anemia, recent use of antibiotics, and recent use of proton-pump inhibitors. In the validation cohort, the sensitivity, specificity, and positive and negative predictive values of the prediction rule, based on a cut-off value of ≥ 0.45, were 78.3%, 70.8%, 29.5%, and 95.4%, respectively.

**Conclusion:**

This clinical prediction rule for identifying toxigenic *C. difficile* carriage at admission may facilitate the selective screening of high-risk groups. To implement it in a clinical setting, more patients from other medical institutions need to be prospectively examined.

## Introduction

In 2013, the United States Centers for Disease Control and Prevention designated *Clostridioides difficile (C. difficile)* as a dangerous pathogen that requires diligent monitoring and prevention activities [1]. Although *C. difficile* infections (CDIs) have traditionally been considered to affect patients in healthcare facilities, the disease epidemiology seems to have shifted, with patients now presenting with community-onset CDI [2].

The clinical severity of CDIs ranges from an asymptomatic carrier state to life-threatening conditions [3]. Asymptomatic carriers may serve as significant reservoirs for transmission to susceptible patients and the environment via direct or indirect contact. They are also six times more likely to develop subsequent symptomatic CDI than non-carriers [4, 5].

Older age (≥ 65 years) is a crucial contributor to CDI development and severity because of age-related immunosenescence, an increase in the use of antibiotics, and frequent exposure to medical environments [6]. Furthermore, previous epidemiological studies revealed that one out of three CDIs and two out of three healthcare-associated CDIs develop in patients aged 65 years or older. Advanced age is also significantly associated with CDI recurrence [7]. However, data on the prevalence and risk factors for toxigenic *C. difficile* carriage on hospital admission in older populations are limited.

Nucleic acid amplification testing (NAAT) is the only diagnostic test for the detection of toxigenic *C. difficile* used in many studies and may result in CDI overdiagnosis [8]. NAAT screening tools for CDI are, however, widely implemented because asymptomatic toxigenic *C. difficile* carriage is a major risk factor for CDI [9].

Early recognition of toxigenic *C. difficile* infection on hospital admission is essential for timely infection control measures to contain the transmission of nosocomial CDI [10–12]. Asymptomatic toxigenic *C. difficile* carriers are also at high risk for progression to symptomatic CDI, for which antimicrobial stewardship measures should be implemented. Therefore, this study aimed to develop and validate a clinical prediction rule to identify older toxigenic *C. difficile* carriers on hospital admission.

## Methods

### Hospital setting

This study was conducted at a 1,048-bed university-affiliated hospital in Seoul, Republic of Korea. Since October 2019, the hospital has run an active surveillance program of toxigenic *C. difficile* carriers, targeted at older patients (≥ 65 years) within 48 h of their admission to the Division of Infectious Diseases, Department of Internal Medicine. As part of the pilot project, a program was implemented to strengthen the antimicrobial stewardship for the targeted population and promote the early detection of symptomatic patients with CDI. However, strict contact isolation, including private room use or cohorting, could not be implemented because of the lack of medical resources.

### Study design

This retrospective cohort study was performed using separate derivative and validation datasets to generate and validate a clinical prediction rule for identifying patients who are toxigenic *C. difficile* carriers at the time of their hospital admission. In the derivation cohort, 1:1 case-control was conducted to identify the risk factors associated with toxigenic *C. difficile* carriage upon hospital admission between October 2019 and April 2021. The formula derived from the multivariable logistic regression analysis was used to establish the clinical prediction rules. A case was defined as an older (≥ 65 years) toxigenic *C. difficile* carrier when confirmed by real-time polymerase chain reaction (PCR) screening using a stool specimen or rectal swab at the time of admission to the Division of Infectious Diseases, Department of Internal Medicine. A control subject was defined as an older patient (≥ 65 years) who did not have toxigenic *C. difficile* at hospital admission. Subsequently, an internal validation was performed on the derived clinical prediction rule in the validation cohort between May 2021 and October 2021.

### Data collection

The following potential predictive variables for toxigenic *C. difficile* carriage or CDI were collected from a computerized hospital database for each patient: age, sex, comorbid conditions, history of procedures or operations over the past month, receipt of proton-pump inhibitors or immunosuppressants, exposure to a medical environment, antibiotic use for more than 3 days over the past month, diagnosis on admission, intensive care unit stay over the past month, and multidrug-resistant microorganisms isolated from clinical specimens during hospitalization. Diarrhea was defined as the passage of three or more loose or liquid stools per day. An asymptomatic carrier was defined as a person infected with *C. difficile*, detected by PCR, without diarrhea.

The study was approved by our hospital’s institutional review board [2022AN0356]. Since the clinical data were obtained through a routine hospital surveillance program for infection control and antimicrobial stewardship, the requirement for informed consent was waived.

### Microbiological methods

Stool samples or rectal swabs were obtained from each patient within 48 h of hospital admission. Toxigenic *C. difficile* carriers were identified with a real-time PCR assay, which simultaneously detects toxin A (TcdA enterotoxin, encoded by *tcdA*) and toxin B (TcdB cytotoxin, encoded by *tcdB*) (AdvanSure CD Real-Time PCR Kit; LG Life Science, Seoul, Korea). Enzyme-linked immunosorbent assay (ELISA) (*C. DIFFICILE* TOX A/B II, TECHLAB, USA) was used to evaluate the stool samples for toxin A and B production.

### Statistical analyses

Existing active surveillance data for toxigenic *C. difficile* carriage were divided into derivation and validation datasets to build a clinical predictive model and validate the clinical performance of the model. The derivation cohort included all carriers of toxigenic *C. difficile* identified at our center between October 2019 and April 2021. A control group was randomly selected from the pool of eligible patients with no toxigenic *C. difficile*, based on a 1:1 pairing. The validation cohort included all subjects who underwent PCR testing for toxigenic *C. difficile* carriage between May 2021 and October 2021. The risk factors for toxigenic *C. difficile* carriage were compared between the case and the control group using Chi-squared tests or Fisher’s exact test for ordinal and dichotomous variables, respectively. Two-sample Student’s *t*-tests or Mann–Whitney *U*-test were used to compare continuous independent variables with normal or non-normal distributions, respectively.

Multivariable logistic regression analysis was conducted using stepwise variable selection based on the Wald statistic criterion. Variables with *P* < 0.05 were included in the final logistic regression model. The Hosmer–Lemeshow goodness-of-fit test was performed to evaluate the final selected model.

A receiver operating characteristic (ROC) curve analysis using the clinical prediction model was conducted to generate a risk index to identify the patients having a higher probability of carrying toxigenic *C. difficile*. The discriminative ability of the models to predict toxigenic *C. difficile* carriage upon hospital admission was assessed through area under the ROC curve (AUC) analysis. The optimal ROC cut-off value was derived from Youden’s index. Furthermore, the performance of the final multivariable logistic regression model was confirmed by evaluating its predictive accuracy using the leave-one-out cross-validation (LOOCV) method and a test dataset. IBM SPSS Statistics, version 20.0 (IBM, Armonk, NY, USA) and SAS 9.4 (SAS Institute Inc., Cary, NC, USA) were used to perform the multivariable logistic regression analysis and to simulate the validation of estimates, respectively.

## Results

### **Prevalence of toxigenic*C. difficile*carriers on hospital admission**

**Fig. 1:**
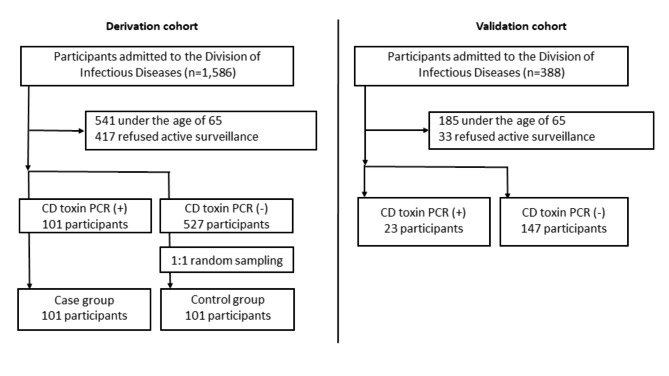
Flow chart illustrating the inclusion of the study participants

During the study period, 1,045 out of the 1,586 patients admitted to the Division of Infectious Diseases of our hospital were of older age (≥ 65 years). PCR screening for toxigenic *C. difficile* carriage was conducted for 628 (60.1%) out of these 1,045 patients (Fig. 1). PCR screening was not performed in the remaining 417 patients because stool samples or rectal swab specimens could not be collected within 48 h of admission or the patient refused to provide rectal swabs or undergo a stool examination.

**Fig. 2:**
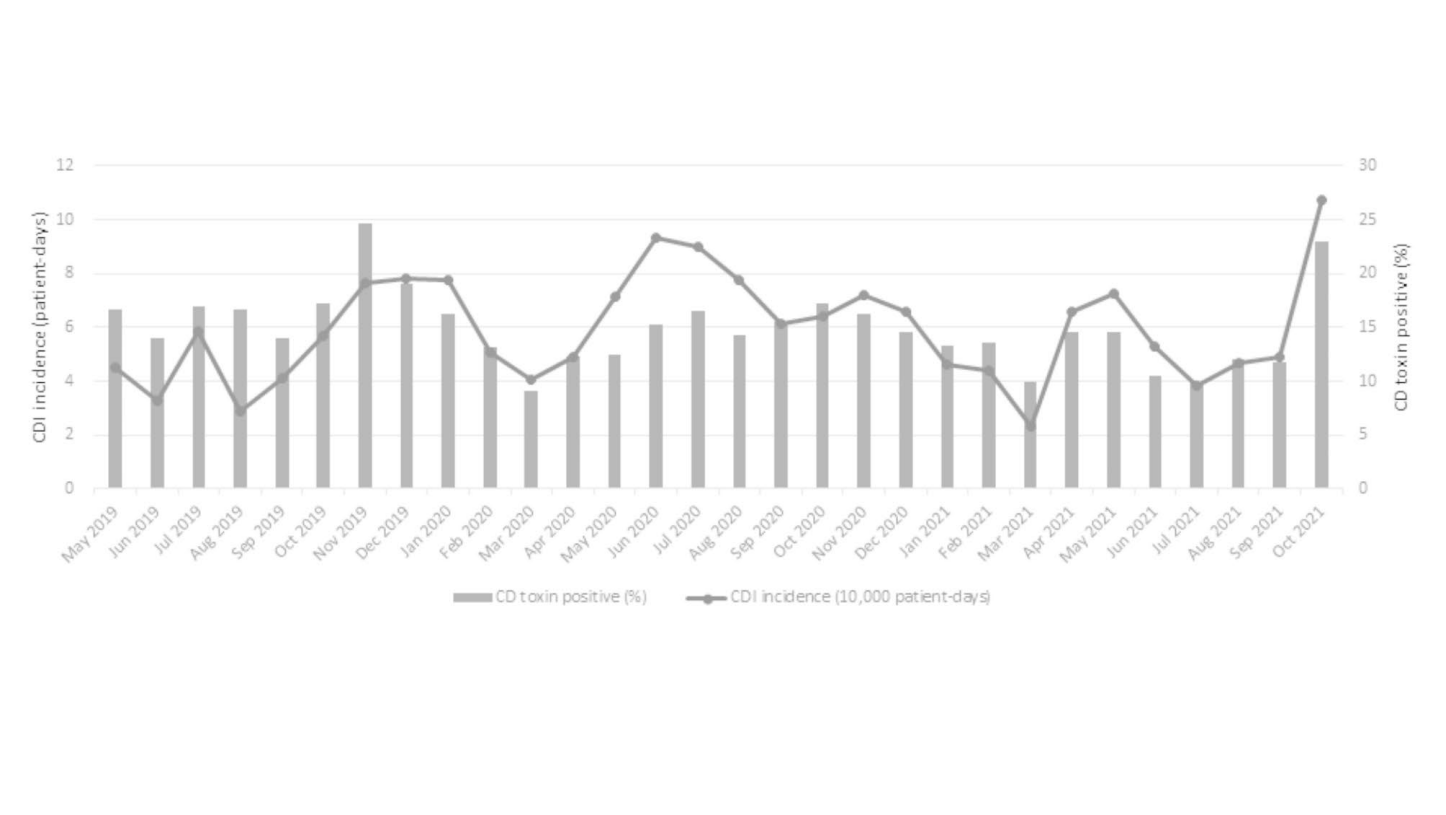
Positivity rates of patients with toxigenic *Clostridioides difficile* during active surveillance and incidence of *C. difficile* carriage at our hospital between May 2019 and October 2021 (CD, *Clostridioides difficile*; CDI, *Clostridioides difficile* infection)

Of the 628 screened patients, 101 (16.1%) were toxigenic *C. difficile* carriers. The monthly prevalence of toxigenic *C. difficile* carriage at admission ranged from 4.0 to 15.9 per 10,000 patient-days, with an average of 8.8 per 10,000 patient-days during the study period (Fig. 2). The demographic and clinical characteristics of the screened patients are shown in Table 1. Of the 101 patients with positive PCR test results, 55 (54.5%) were diagnosed with symptomatic CDI (45 healthcare-associated and 10 community-acquired cases) and underwent antibiotic therapy for CDI. In particular, nine patients were diagnosed with symptomatic CDI at admission, and 24, including these patients, started CDI treatment within 48 h of admission.

**Table 1:** Comparison of demographic and clinical characteristics between toxigenic *Clostridioides difficile* carriers and non-carriers at hospital admission in the derivation group

	Total (n = 202)	CD toxin-negative (n = 101)	CD toxin-positive (n = 101)	*P*-value
**Age (years)**, **median (IQR)**	79 (73–85)	78 (72–84)	80 (74–85)	0.357
**Male, n** (%)	99 (49.0)	47 (46.5)	52 (51.5)	0.482
**Admission route, n (%)**				
Emergency room	182 (90.1)	91 (90.1)	91 (90.1)	1.000
Outpatient clinic	20 (9.9)	10 (9.9)	10 (9.9)	1.000
**History of CDI before admission, n (%)**	3 (0.01)	0 (0)	3 (3.0)	-
**Pre-admission route, n (%)**				< 0.001
Home	104 (51.5)	66 (65.3)	38 (37.6)	< 0.001
Acute care hospital	38 (18.8)	16 (15.8)	22 (21.8)	0.280
Nursing hospital	44 (21.8)	11 (10.9)	33 (32.7)	< 0.001
Nursing facility	16 (7.9)	8 (7.9)	8 (7.9)	1.000
**MDR acquisition during admission (%)**				
CRE	7 (3.5)	2 (2.0)	5 (5.0)	0.248
VRE	20 (9.9)	9 (8.9)	11 (10.9)	0.638
CRAB	29 (14.4)	7 (6.9)	22 (21.8)	0.003
CRPA	15 (7.4)	6 (5.9)	9 (8.9)	0.421
MRSA	42 (20.8)	15 (14.9)	27 (26.7)	0.037
Total MDROs	69 (34.2)	24 (23.8)	45 (44.6)	0.002
**Infectious diseases at the time of admission, n (%)**				
Urinary tract infections	132 (65.3)	57 (56.4)	75 (74.3)	0.008
Pneumonia	95 (47)	39 (38.6)	56 (55.4)	0.017
Skin and soft tissue infections	16 (7.9)	9 (8.9)	7 (6.9)	0.602
Central nervous system infections	6 (3)	1 (1.0)	5 (5.0)	0.097
Bone and joint infections	17 (8.4)	3 (3.0)	14 (13.9)	0.005
Intra-abdominal infections	68 (33.7)	29 (28.7)	39 (38.6)	0.137
Septic shock	58 (28.7)	18 (17.8)	40 (39.6)	0.001
**Comorbidities (%)**				
Cardiovascular diseases	157 (77.7)	79 (78.2)	78 (77.2)	0.866
Neurologic diseases	107 (52.9)	46 (45.5)	61 (60.4)	0.034
Malignant diseases	39 (19.3)	19 (18.8)	20 (19.8)	0.859
Trauma	26 (12.8)	11 (10.9)	15(14.9)	0.401
Chronic renal diseases	61 (30.1)	36 (35.6)	25 (24.8)	0.092
Chronic liver diseases	26 (12.8)	12 (11.9)	14 (13.9)	0.674
Chronic pulmonary diseases	63 (31.1)	23 (22.8)	40 (39.6)	0.010
Connective tissue diseases	9 (4.4)	1 (1.0)	8 (7.9)	0.017
Metabolic diseases	101 (5)	48 (47.5)	53 (52.5)	0.482
Hematologic diseases	145 (71.7)	60 (59.4)	85 (84.2)	< 0.001
Charlson’s comorbidity score, median (IQR)	3 (2–4)	4 (3–5)	2 (1–4)	< 0.001
**Predisposing factors within 30 days, n (%)**				
Recent surgery	16 (7.9)	4 (4.0)	12 (11.9)	0.037
Recent admission	93 (46)	29 (28.7)	64 (63.4)	< 0.001
Use of corticosteroids	36 (17.8)	16 (15.8)	20 (19.8)	0.462
Use of antibiotics	97 (48)	30 (29.7)	67 (66.3)	< 0.001
Intensive care unit stay	35 (17.3)	13 (12.9)	32 (31.7)	0.001
Foley catheterization	85 (42)	29 (28.7)	56 (55.4)	< 0.001
Central venous catheterization	43 (21.2)	15 (14.9)	28 (27.7)	0.025
Nasogastric tube	61 (30.1)	21 (20.8)	40 (39.6)	0.004
Percutaneous drainage,	29 (14.3)	11 (10.9)	18 (17.8)	0.160
Mechanical ventilation	17 (8.4)	4 (4.0)	13 (12.9)	0.023
Tracheostomy	16 (7.9)	6 (5.9%)	10 (9.9%)	0.297
Hemodialysis	7 (3.4)	1 (1.0)	6 (5.9)	0.054
Bed-ridden status	78 (38.6)	28 (27.7)	50 (49.5)	0.001
Sore sites	67 (33.1)	21 (20.8)	46 (45.5)	< 0.001
Proton-pump inhibitors	95 (47)	31 (30.7)	64 (63.4)	< 0.001
Use of probiotics	80 (39.6)	21 (20.8)	59 (58.4)	< 0.001
Anemia (hemoglobin ≤ 10 g/dL)	131 (64.9)	29 (28.7%)	64 (63.4)	< 0.001
**Length of hospital stay (days)**, **median (IQR)**	14 (9–24)	11 (7–18)	16 (10–28)	0.236
**In-hospital mortality, n (%)**	14 (6.9)	6 (5.9)	8 (7.9)	0.580

CD, *Clostridiodes difficile*; CDI, *Clostridiodes difficile* infection; CRAB, carbapenem-resistant *Acinetobacter baumannii*; CRE, carbapenem-resistant Enterobacteriaceae; CRPA, carbapenem-resistant *Pseudomonas aeruginosa*; IQR, interquartile range; MDR, multidrug-resistant; MDROs, multidrug-resistant microorganisms; MRSA, methicillin-resistant *Staphylococcus aureus*; VRE, vancomycin-resistant enterococci

### Construction of the clinical prediction model

Carriers of toxigenic *C. difficile* among our screened patients were more likely to reside in a facility than at home and be exposed to medical procedures 30 days before admission than those who were non-carriers (Table 1). Carriers had more neurological, chronic pulmonary, and connective tissue as well as hematologic diseases than non-carriers (Table 1). Only three patients with toxigenic *C. difficile* had a history of CDI 90 days before admission. Multidrug-resistant microorganisms, such as carbapenem-resistant *Acinetobacter baumannii* or methicillin-resistant *Staphylococcus aureus* were more commonly isolated from clinical samples during hospitalization in carriers than in non-carriers (Table 1).

In the multivariable logistic regression model, septic shock, connective tissue diseases, anemia, recent use of antibiotics, and recent use of proton-pump inhibitors were significant risk factors associated with toxigenic *C. difficile* carriage at the time of admission in older patients admitted to the Division of Infectious Diseases, Department of Internal Medicine (Table 2).

**Table 2:** Multivariable logistic regression analysis of the risk factors associated with toxigenic *Clostridioides difficile* carriage at hospital admission in the derivation group (final model)

Risk factors	ß-coefficient	Standard error	Odds ratio	95% confidence interval	*P*-value
**Intercept**	-1.93	0.35			< 0.0001
**Septic shock**	0.87	0.39	2.39	1.13–5.09	0.0234
**Connective tissue diseases**	3.03	1.14	20.64	2.23–191	0.0077
**Anemia**	0.87	0.36	2.39	1.18–4.85	0.0161
**Use of antibiotics**	1.39	0.34	4.03	2.07–7.86	< 0.001
**Use of proton-pump inhibitors**	0.72	0.34	2.06	1.05–4.05	0.0355

We calculated the predictive probability of patients being toxigenic *C. difficile* carriers using the following formula:

$$\begin{array}{l}{\rm{P(Y = yes)}}\\{\rm{ = }}\frac{{{\rm{exp( - 1}}{\rm{.93 + 0}}{\rm{.87A + 3}}{\rm{.03B + 0}}{\rm{.87C + 1}}{\rm{.39D + 0}}{\rm{.72E)}}}}{{{\rm{1 + exp( - 1}}{\rm{.93 + 0}}{\rm{.87A + 3}}{\rm{.03B + 0}}{\rm{.87C + 1}}{\rm{.39D + 0}}{\rm{.72E)}}}}\end{array}$$

A: If septic shock, positive = 1 / negative = 0; B: If connective tissue diseases, positive = 1 / negative = 0; C: If anemia, positive = 1 / negative = 0; D: If recent use of antibiotics, positive = 1 / negative = 0; and E: If recent use of proton-pump inhibitors, positive = 1 / negative = 0.

When a cut-off value of ≥ 0.45 was applied to the clinical prediction model, the AUC value was 0.80, with a 95% confidence interval (CI) of 0.74–0.86 in the derivation cohort. The sensitivity, specificity, positive predictive value, and negative predictive value of this prediction rule were 75.3% (95% CI, 65.7–83.3), 74.3% (95% CI, 64.6–82.4), 74.5% (95% CI, 67.3–80.6), and 75.0% (95% CI: 67.7–81.1), respectively (Table 3). As shown in Table 3, the test and training data sets had similar accuracy, sensitivity, and specificity. The Hosmer–Lemeshow goodness-of-fit test result for the final model was *P* = 0.58, yielding no evidence for a lack of fit.

**Table 3:** Area under the ROC curve and probability of toxigenic *Clostridioides difficile* carriage at hospital admission based on a cut-off value in the derivation population, with cross-validation, and in the validation cohort

Variable	AUC ± SE	95% CI	Cut-off value	Sensitivity	95% CI	Specificity	95% CI	PPV	95% CI	NPV	95% CI
**Training dataset**	0.80 ± 0.031	0.74–0.86	0.45	75.25	65.7–83.3	74.3	64.6–82.4	74.5	67.3–80.6	75.0	67.7–81.1
**Cross-validation (training dataset)**	0.76 ± 0.035	0.70–0.82	0.47	75.25	65.7–83.3	74.3	64.6–82.4	74.5	67.3–80.6	75.0	67.7–81.1
**Test dataset**	0.84 ± 0.035	0.78–0.90	0.45	78.26	56.3–92.5	70.8	62.7–78.0	29.5	23.1–36.8	95.4	90.5–97.8

AUC, area under the receiver operating characteristic curve; CI, confidence interval; NPV, negative predictive value; PPV, positive predictive value; SE, standard error

### Validation of the clinical prediction model

The validation cohort included 170 patients who underwent PCR screening upon admission for toxigenic *C. difficile* carriage between April 2021 and October 2021. PCR screening was not performed in 33 patients because stool samples or rectal swab specimens could not be collected within 48 h of admission. This prediction risk model showed an AUC of 0.84 (95% CI, 0.78–0.90) in the validation dataset. The sensitivity, specificity, positive predictive value, and negative predictive value of this prediction rule were 78.3% (95% CI, 56.3–92.5), 70.75% (95% CI, 62.7–78.0), 29.5% (95% CI, 23.1–36.8), and 95.4% (95% CI, 90.5–97.8), respectively (Table 3). Of the 170 screened patients, 23 were positive for *C. difficile* toxin and 147 were negative. Our prediction rule confirmed that 27 were false positives and six were false negatives.

## Discussion

In this study, we generated a clinical prediction rule for identifying patients with toxigenic *C. difficile* at hospital admission through active surveillance of older patients (≥ 65 years old) hospitalized with infectious diseases with a high probability of using antibiotics. Surprisingly, our analyses show that the prevalence of toxigenic *C. difficile* carriage on admission was as high as 16.1%. Considering the high cost of PCR testing, this scoring system may be valuable for selective screening to reduce the screening volumes at our hospital.

Previous studies have reported that *C. difficile* carriage prevalence rates in healthcare settings among the older population range from 1.6% for patients in the community to 21% for those in short- or long-term care facilities [13]. In our study, the 16.1% prevalence of toxigenic *C. difficile* carriage on admission was acceptable, considering that only 51.5% of the older patients were admitted without exposure to other healthcare facilities. These prevalence rates may vary according to the type of medical institution and surveillance methods used. Previous studies have reported a *C. difficile* carriage prevalence among older adults on admission of approximately 10% in culture-based and 16.4% in PCR-based screenings [14, 15]. Commercial PCR assays can provide a rapid and sensitive alternative to sample culture screenings for *C. difficile*, despite their high cost and rising concerns about false positivity.

We found that 50.5% (46/92) of asymptomatic toxigenic *C. difficile* carriers developed symptoms of CDI during hospitalization. A previous study demonstrated that toxigenic *C. difficile* carriers identified in an active screening had a 23-fold greater risk of developing CDI than non-carriers [16]. Other studies have also suggested that 2 − 37% of asymptomatic *C. difficile* carriers become symptomatic [17, 18].

In our study, septic shock, connective tissue diseases, anemia, recent use of antibiotics, and recent use of proton-pump inhibitors were significant risk factors associated with toxigenic *C. difficile* carriage at the time of admission in older patients with infectious diseases. Similar to our findings, previous studies have identified old age, underlying diseases, prior hospitalization, low Norton scores, pressure sores, and recent use of antibiotics, proton-pump inhibitors, and corticosteroids as independent risk factors for *C. difficile* colonization or infection [19–22]. In the present study, anemia and septic shock were significant predictors of toxigenic *C. difficile* carriage among older individuals. These conditions compromise the immune function and may be associated with underlying chronic diseases. A recent study suggested that anemia with hemoglobin levels < 10 g/dL is a risk factor associated with poor CDI outcomes [23]. Furthermore, iron appears to contribute to *C. difficile* colonization and CDI pathogenesis in mouse models [24]. Although there is no convincing evidence linking septic shock with toxigenic *C. difficile* carriage, CDI may develop during antibiotic treatment for worsening sepsis caused by a separate bacterial infection.

Our study provides a clinical model to predict toxigenic *C. difficile* carriage in older patients. However, previous studies have developed clinical rules to predict primary CDI onset, as well as CDI recurrence, complications, and mortality, with a sensitivity of 60 − 98% and a specificity of 44 − 95% [25–29]. The variables included in the clinical prediction rules for predicting the primary CDI onset were heterogeneous, such as old age, recent-onset diarrhea, development of infection during a prior admission, residing in a long-term care facility, admission to an intensive care unit, length of stay of 7 days or longer, endoscopy within 30 days, recent use of broad-spectrum antibiotics such as cephalosporins or fluoroquinolones, use of laxatives, gastric acid suppressors, or antimotility drugs, low body mass index (< 25), hypoalbuminemia, CDI pressure, and hemodialysis [25–28].

Asymptomatic *C. difficile* carriage is well known as a major risk factor for developing symptomatic CDI. Furthermore, although asymptomatic carriers pose a substantial reservoir for the transmission of CDI, control measures focus almost entirely on symptomatic patients [30]. While the current guidelines do not recommend active surveillance or the isolation of asymptomatic *C. difficile* carriers as measures to prevent *C. difficile* transmission, several studies have shown that active screening for *C. difficile* carriage using PCR assays is effective for infection control and prevention of *C. difficile* infection [10–12]. Ongoing research on new strategies, such as screening for asymptomatic *C. difficile* carriage, is needed to optimize the containment of these infections.

This study has a few limitations. First, this was a retrospective, single-center study that used random sampling of control cases. The study participants were limited to patients at our hospital. Therefore, a longitudinal prospective validation of the performance of this model in an external population is needed. Afterwards, an intervention study is needed to evaluate the effect of infection control or antimicrobial stewardship programs, including a screening tool for *C. difficile* carriage. Second, screening compliance was not universal. Because the unscreened patients might be less frail or sick than the screened patients, a selection bias might have occurred and, as a result, toxigenic *C. difficile* carriage might have been overestimated in our datasets. Third, owing to the small number of study subjects in our analysis, it was not possible to identify the risk factors that differentiate between asymptomatic *C. difficile* carriers and symptomatic patients with CDI on admission. Finally, PCR assays rather than cultures were used to identify toxigenic *C. difficile* carriers; the former may, however, be oversensitive in detecting *C. difficile* toxins.

## Conclusion

Consistent with those of recent reports, our findings report that older patients with toxigenic *C. difficile* at hospital admission are common. Thus, our clinical prediction rule, as an initial screening tool, followed by PCR screening for prediction rule-positive patients, could reduce the PCR screening volumes. However, clinical prediction rules specific to local hospitals should be periodically verified, and these strategies should be fully integrated into existing infection control programs that include thorough contact precautions, cohorts, and environmental disinfection.

## Data Availability

The data that support the findings of this study are available on request from the corresponding author.

## Abbreviations

AUC: Area under the curve
CD: *Clostridioides difficile*
CDI: *Clostridioides difficile* infection
CRAB: Carbapenem-resistant *Acinetobacter baumannii*
CRE: Carbapenem-resistant Enterobacteriaceae
CRPA: Carbapenem-resistant *Pseudomonas aeruginosa*
CVC: Central venous catheter
ER: Emergency room
MDROs: Multi-drug resistant organisms
MRSA: Methicillin-resistant Staphylococcus aureus
PCR: Polymerase chain reaction
PPI: Proton-pump inhibitor
VRE: Vancomycin-resistant enterococci
